# A graph clustering algorithm with hypergraph learning and a core-attachment strategy for protein complex identification

**DOI:** 10.3389/fgene.2026.1770432

**Published:** 2026-03-30

**Authors:** Jie Wang, Xiancan Yang, Pengbo Yang, Jian Yang, Yangyang Miao

**Affiliations:** School of Information, Shanxi University of Finance and Economics, Taiyuan, China

**Keywords:** graph clustering, graph embedding, hypergraph learning, protein complexes, protein-protein interaction networks

## Abstract

Protein complexes play a crucial role in cellular biological processes. Identifying these complexes is essential for understanding cellular functions and biological mechanisms. Graph clustering approaches to identify protein complexes in protein-protein interaction (PPI) networks have become a significant research hotspot in data mining and bioinformatics. Many graph clustering methods have been developed for protein complex identification. However, most existing methods only utilize original networks to discover dense subgraphs and ignore higher-order topological characteristics. Considering the prevalent multi-relational and complex interactions in biological networks, a graph clustering algorithm based on hypergraph learning and a core-attachment strategy is proposed for protein complex identification, called HLCA. Hypergraph networks are employed to directly model multi-relational interactions. Based on this method, a multi-level hypergraph is used as higher-order topology and a core-attachment strategy are adopted to identify protein complexes. Firstly, the original PPI network is transformed into a hypergraph network. Secondly, a hierarchical compression strategy is applied to recursively compress the hypergraph into smaller hypergraphs at various levels, forming a multi-level analytical framework. Thirdly, hypergraph convolution is performed across different hierarchical levels to obtain node representations at each level. These node representations are then combined to produce complete node embeddings. Based on these node embeddings, a weighted PPI network is constructed by cosine similarity from the original PPI network. Core clusters are obtained in this weighted network by cluster density. Finally, remaining protein nodes are added to the core clusters using a core-attachment strategy combining hyperedge density and overlap. The effectiveness of HLCA is evaluated by comparing it with other protein complex identification methods on multiple datasets. Experimental results show that the proposed method outperforms comparison methods regarding F-measure and Accuracy.

## 1 Introduction

Proteins are large organic molecules formed by amino acids linked through peptide bonds. They serve as core executors of cellular structure and function. Proteins participate in nearly all biological processes, including enzyme catalysis, signal transduction, and immune defense. However, most proteins do not function individually. Instead, they form complexes with other biomolecules such as proteins and nucleic acids to perform complex biological functions (Zhang et al., 2015). As critical functional units in cellular processes, protein complexes participate in numerous biological activities, including cell cycle regulation, signal transduction, and gene expression regulation. Extensive evidence indicates that protein complexes are closely associated with various diseases (Wu et al., 2013), making them significant for biomedical research.

Experimental methods for identifying protein complexes, such as nuclear magnetic resonance (NMR) (Göbl et al., 2014) and mass spectrometry (Walzthoeni et al., 2013), are costly and time-consuming. Thus, they are unable to comprehensively identify all protein complexes. Computational methods identify protein complexes by mining closely connected protein node sets in protein-protein interaction (PPI) networks (Alberts, 1998). PPIs can be obtained using sequence-based prediction methods (Dunham and Ganapathiraju, 2021), deep learning-based prediction methods (Hua et al., 2022; Cao and Chen, 2023), and evolutionary relationship-based prediction methods (Li et al., 2008b). These methods help construct comprehensive PPI networks. Developing computational methods based on network topology to identify protein complexes from PPI networks has become a major research direction. Such methods contribute to understanding complex intracellular biological processes, promote disease research, and facilitate drug discovery, highlighting their significant research value and application potential (Pan et al., 2022). More details on the related work are introduced in the related work section.

Currently, numerous computational methods have been proposed for protein complex identification. Among them, the methods that utilize network topology information mainly include graph partitioning algorithms, heuristic-based algorithms, and graph embedding algorithms, etc.

The core idea of graph partitioning algorithms is to optimize a specific objective function. These methods partition a graph into multiple disjoint subgraphs that contain a certain number of nodes or edges. Connections within subgraphs are dense, while connections between subgraphs are sparse (Shang et al., 2025). Algorithms based on this idea usually consider each subgraph as a protein complex. For example, the Markov clustering algorithm (MCL) (Vlasblom and Wodak, 2009) identifies subgraphs by simulating random walks. The algorithm adds loops to the input graph and transforms it into a Markov matrix. This matrix is repeatedly updated through expansion and inflation operations until the graph is segmented into multiple subgraphs. Each subgraph represents a predicted protein complex. However, this approach produces only non-overlapping clusters. Thus, although MCL can handle noisy nodes, it cannot detect overlapping clusters. The Soft Regularized MCL (SR-MCL) algorithm (Shih and Parthasarathy, 2012) was proposed to address this limitation. Algorithms developed on this concept, such as BOPS (Lyu et al., 2022) and DPFO (Wang et al., 2025a), further improved clustering performance. Protein complex identification algorithms developed under this approach perform complex identification by partitioning PPI networks. Such methods neglect the authentic multi-node cooperative interactions among protein populations and therefore fail to capture the many-to-many interaction characteristics intrinsic to biological networks.

Heuristic-based algorithms identify protein complexes by selecting seed proteins as initial clusters and then greedily expanding these clusters. Classic examples include ClusterONE (Nepusz et al., 2012) and DPClus (Altaf-Ul-Amin et al., 2006). ClusterONE addresses overlapping clusters in PPI networks. It starts from individual seed nodes and applies a greedy strategy to identify high-density clusters. ClusterONE merges overlapping clusters by quantifying the overlap between each pair and discarding clusters that do not meet certain criteria. It treats high-density regions as protein complexes. DPClus introduces the concept of “cluster periphery”. It assigns edge weights based on common neighbors and determines node weights from the sum of adjacent edge weights. DPClus starts clustering from the node with the highest weight and expands the cluster iteratively until the density and cluster-periphery conditions are satisfied. DPClus accurately identifies high-density regions. Another heuristic approach based on seed expansion is the core-attachment method. Gavin et al. (Gavin et al., 2006) initially proposed that protein complexes consist of core and attachment components. The main idea is to divide protein complexes into two functional units: core proteins and attachment proteins. Core proteins are stable and frequently recurring protein groups, while attachment proteins are loosely connected proteins dynamically associating with or dissociating from the core. Based on this concept, the CORE algorithm (Leung et al., 2009) introduces the core-attachment model. It predicts core components and identifies attachment proteins interacting with these cores. The COACH method (Wu et al., 2009) selects proteins whose node degrees are above average as preliminary core proteins within their neighborhood. A recursive core-elimination method extracts high-density subgraphs as the final cores. Attachment proteins are identified from neighboring nodes that interact with more than half of the core proteins. Attachment proteins are then combined with core proteins to form predicted protein complexes. Based on these approaches, further algorithms such as WPNCA (Peng et al., 2014), WCOACH (Kouhsar et al., 2015), SEGC (Wang et al., 2017), GCAPL (Wang et al., 2023), and Multiobjective (Mukhopadhyay et al., 2024) have been developed. Heuristic protein complex identification algorithms operate on PPI networks by modeling one-to-one node interactions. Such designs similarly neglect the complex many-to-many interaction characteristics intrinsic to biological networks.

Graph embedding is a technique that maps nodes (or other graph structures such as edges and subgraphs) into low-dimensional vector spaces. This mapping preserves structural information from the original topological graph. Similarities between nodes in the embedding space approximate their relationships in the original network (Xu, 2021). For example, DPCMNE (Meng et al., 2021), captures local and global topological information by embedding nodes into low-dimensional vector spaces at different granularities. Initially, the Louvain algorithm divides the original PPI network into modules by grouping closely connected protein nodes. For these modules, the DeepWalk algorithm obtains low-dimensional embeddings of proteins. These embeddings are then used to construct weighted networks, and protein complexes are subsequently identified using a core-attachment strategy. Another algorithm, ELF-DPC (Wang et al., 2022) proposes an ensemble learning framework. This framework integrates various types of information and employs voting regression models to enhance protein complex identification. Furthermore, hypergraph models grounded in graph embedding concepts have been introduced and combined with diverse bioinformatics data and protein dynamic features for protein complex identification, exemplified by HyperGraphComplex (Xia et al., 2024) and HGST (Wang et al., 2025b). Most graph embedding-based protein complex identification algorithms project PPI networks into low-dimensional vector spaces. This projection neglects higher-order non-pairwise interactions among protein nodes.

Most existing protein complex identification methods rely on pairwise interactions derived from PPI networks. Such low-order modeling approaches cannot accurately capture higher-order cooperative interactions among protein groups. To overcome this limitation, hypergraph-based modeling has been introduced to represent complex structural interactions in biological networks. Unlike traditional graphs that restrict relationships to pairs of nodes, hypergraphs allow a single hyperedge to connect multiple nodes simultaneously, thereby enabling the modeling of higher-order and non-pairwise interactions among proteins (Gao et al., 2022). This one-to-many relationship representation is particularly suitable for describing protein complexes composed of multiple interacting proteins.

In summary, this paper propose a graph clustering algorithm for protein complex identification based on hypergraph learning and a core-attachment strategy, named HLCA. The HLCA algorithm transforms the PPI network into a hypergraph and extracts topological features at multiple scales through a hierarchical compression strategy, resulting in informative protein node embeddings. Protein complexes are then identified using these embeddings in combination with a core-attachment strategy. This approach overcomes the limitations of traditional graph-based pairwise interaction modeling and effectively integrates both local and global topological information from PPI networks, providing a novel and powerful framework for protein complex identification.

## 2 Methods

**FIGURE 1:**
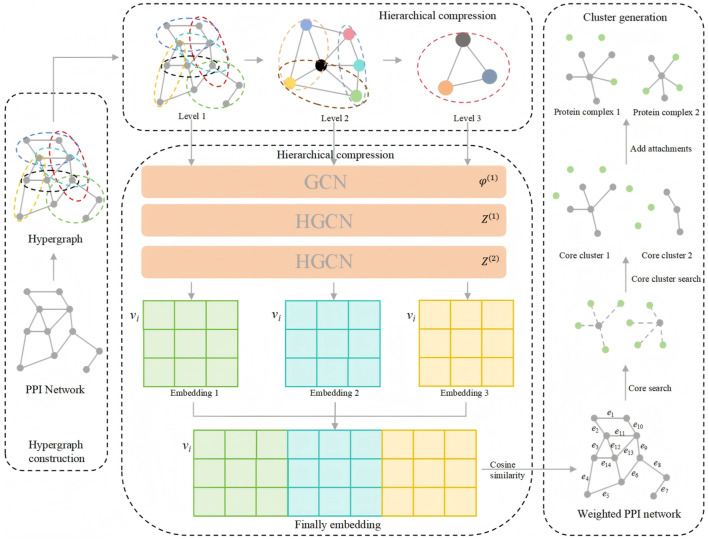
The workflow of the proposed HLCA algorithm.

The HLCA algorithm consists of four major components: hypergraph construction, hierarchical compression, node embedding, and cluster generation. First, the original PPI network is converted into a hypergraph. Second, during the hierarchical compression phase, a hypergraph modularity-based strategy is employed to recursively compress the hypergraph into multiple smaller hypergraphs at different levels. Third, in the node embedding phase, hypergraph convolution operations are applied to these compressed hypergraphs across all hierarchical levels, generating multi-scale embedding representations for each node. These representations are subsequently integrated to obtain a final unified node embedding vector. Finally, in the cluster generation phase, a weighted PPI network is constructed by computing the cosine similarity between node embeddings. Core clusters are then identified within this weighted network based on cluster density. Additional protein nodes are subsequently assigned to the core clusters using a core-attachment strategy that incorporates both hyperedge density and overlap. The complete workflow of the HLCA algorithm is illustrated in Figure 1.

### 2.1 Hypergraph construction

The PPI network is typically modeled as an undirected graph $G=\left(V_{\text{ppi}},E_{\text{ppi}}\right)$ . In this work, we transform the PPI network into a hypergraph denoted by G=(V,E,W,X) , where V is the set of nodes, E is the set of hyperedges, W is a diagonal matrix of hyperedge weights, and $X\in R^{n\times q}$ represents the node feature matrix. Here, n is the number of nodes and q is the dimensionality of node features.

To construct the hypergraph, we define the neighborhood of each protein node $v_{i}$ in the PPI network as Equation 1:

$$N\left(v_{i}\right)=\left\{\;v_{j}\in V_{\text{ppi}}|\left(v_{i},v_{j}\right)\in E_{\text{ppi}}\;\right\}$$ (1)

where $\left(v_{i},v_{j}\right)\in E_{\text{ppi}}$ indicates an interaction between proteins $v_{i}$ and $v_{j}$ in the original PPI network. Based on this definition, the hyperedge associated with node $v_{i}$ is constructed as Equation 2:

$$e_{k}=\left\{v_{i}\right\}\cup N\left(v_{i}\right)$$ (2)

which represents the local interaction group centered at protein $v_{i}$ . When two nodes mutually interact, the hyperedges they induce are equivalent; thus, $e_{k}=\left\{v_{i},v_{j}\right\}$ and $e_{k^{\prime }}=\left\{v_{j},v_{i}\right\}$ denote the same hyperedge.

The incidence matrix $H\in R^{\left|V|\times |E\right|}$ characterizes the relationship between nodes and hyperedges. Each element $h\left(v_{i},e_{k}\right)$ indicates whether node $v_{i}$ belongs to hyperedge $e_{k}$ , and is defined in Equation 3:

$$h\left(v_{i},e_{k}\right)=\left\{\begin{array}{cc} 1, & \text{if }v_{i}\in e_{k}\\ 0, & \text{otherwise} \end{array}\right.$$ (3)

The hyperedge degree matrix is defined as $D_{e}=diag\left(D_{e_{1}},\ldots ,D_{e_{k}}\right)$ , where $D_{e_{k}}$ represents the number of nodes contained in hyperedge $e_{k}$ . The node degree matrix is defined as $D_{v}=diag\left(D_{v_{1}},\ldots ,D_{v_{i}}\right)$ , where $D_{v_{i}}$ reflects the weighted degree of node $v_{i}$ in the hypergraph. The hyperedge degree and node degree are computed in Equation 4 and Equation 5, respectively:

$$D_{e_{k}}=\underset{v_{i}\in V}{\sum }h\left(v_{i},e_{k}\right)$$ (4)

$$D_{v_{i}}=\underset{e_{k}\in E}{\sum }\omega \left(e_{k}\right)\;h\left(v_{i},e_{k}\right)$$ (5)

where $\omega \left(e_{k}\right)=1/|e_{k}|$ denotes the weight of hyperedge $e_{k}$ , and $\left|e_{k}\right|$ is the number of nodes it contains. This method mitigates the bias introduced by large hyperedges and ensures a balanced representation of local structural information in the hypergraph.

### 2.2 Hierarchical compression

In this section, a hypergraph modularity method is employed to partition the hypergraph G into smaller hypergraphs. Through hierarchical compression, both local and global topological information of the hypergraph can be effectively extracted (Kumar et al., 2020).

Traditional hypergraph modularity introduces a null model and defines modularity directly based on this model. However, in biological networks, hyperedges derived from PPI networks often vary substantially in size. If the traditional method is applied directly, large hyperedges may dominate the model. Direct use of the traditional criterion may overemphasize large hyperedges and reduce the validity of hierarchical compression. To mitigate this limitation, a node-degree–preserving hypergraph modularity (Kumar et al., 2020) is adopted. The hypergraph is converted into a weighted graph, and node degrees are preserved during the conversion. This step reduces degree bias caused by large hyperedges. A null model is then defined on the weighted graph to obtain a revised modularity measure. Hierarchical compression is performed through this method, which also enables the extraction of both local and global topology. The multi-level embedding strategy allows HLCA to effectively maintain both fine-grained local structural details and higher-order connectivity patterns by integrating representations from both low-order and high-order embeddings.

Under this formulation, the hypergraph is transformed into a weighted graph. Each hyperedge $e_{k}$ is replaced by a clique formed by all of its protein nodes. The resulting weighted adjacency matrix is defined in Equation 6:

$$A^{\text{clique}}=HWH^{T}$$ (6)

where H is the incidence matrix of the hypergraph and W is the diagonal matrix of hyperedge weights. After removing self-loops, the degree of protein node $v_{i}$ in the weighted graph is computed in Equation 7:

$$\deg_{v_{i}}=\underset{j}{\sum }A_{ij}^{\text{clique}}=\underset{e_{k}\in E}{\sum }h\left(v_{i},e_{k}\right)\;\omega \left(e_{k}\right)\;\left(D_{e_{k}}-1\right)$$ (7)

To ensure that the node degrees of the weighted graph align with those of the original hypergraph, a normalized weighted adjacency matrix is defined in Equation 8:

$$A^{\text{hyp}}=H\cdot W\cdot {\left(D_{e}-I\right)}^{-1}\cdot H^{T}$$ (8)

where ${\left(D_{e}-I\right)}^{-1}$ normalizes the contribution of each hyperedge.

A degree-preserving null model is then introduced, and the expected interaction weight between nodes is computed in Equation 9:

$$P_{ij}^{\text{hyp}}=\frac{D_{v_{i}}\times D_{v_{j}}}{\sum_{v\in V}nd\left(v\right)}$$ (9)

where $\sum_{v\in V}nd\left(v\right)$ represents the total weighted degree of all nodes in the original hypergraph.

The hypergraph modularity matrix is obtained as shown in Equation 10.

$$B_{ij}^{\text{hyp}}=A_{ij}^{\text{hyp}}-P_{ij}^{\text{hyp}}$$ (10)

Using this matrix, the hypergraph modularity value is computed using Equation 11:

$$Q^{\text{hyp}}=\frac{1}{2m}\underset{ij}{\sum }B_{ij}^{\text{hyp}}\;\delta \left(v_{i},v_{j}\right)$$ (11)

where $\delta \left(v_{i},v_{j}\right)=1$ if $v_{i}$ and $v_{j}$ belong to the same community, and 0 otherwise. m denotes the total weight of all edges in the weighted graph.

For a given hypergraph G , each node is initially assigned to its own community. During optimization, the modularity gain $\Delta Q^{\text{hyp}}$ is evaluated for each possible community reassignment. A node is moved only if the reassignment increases $Q^{\text{hyp}}$ ; otherwise, it remains in its current community. When no further improvement is possible, the layer is considered converged.

The hierarchical compression process consists of modularity optimization and module aggregation. The hypergraph modularity $Q^{\text{hyp}}$ divides the hypergraph G into several modules. Each module is aggregated as a “supernode” at this stage to form a new and smaller hypergraph $G_{1}$ . Next, modularity optimization is reapplied to the new hypergraph $G_{1}$ . This iterative process achieves hierarchical compression and produces a series of multi-level compressed hypergraphs $G_{\text{all}}=\left\{G_{1},G_{2},\ldots ,G_{\text{he}}\right\}$ . From the perspective of the higher-order hypergraph, local and global topological information can be analyzed. The embedding representations of protein nodes at different scales are learned.

### 2.3 Node embedding

Hypergraph convolution is applied to all hypergraphs $G_{\text{all}}=\left\{G_{1},G_{2},\ldots ,G_{\text{he}}\right\}$ obtained through hierarchical compression to learn protein node embeddings. The original topological graph considers only first-order proximity, which limits its ability to capture complex relationships among nodes. As a result, the derived adjacency matrix is sparse, hindering effective node embedding in the hypergraph. To address this limitation, an attribute similarity graph is constructed to uncover latent associations between protein nodes. This enriched connectivity facilitates more effective hypergraph convolution and improves representation learning. The adjacency matrix of the attribute similarity graph is denoted by S , where each entry is defined in Equation 12:

$$S=\left[x_{i}^{T}x_{j}\right]$$ (12)

where $x_{i}={\left(A_{ij}\right)}_{j\in V_{\text{ppi}}}$ . The adjacency matrix of the original PPI network is defined in Equation 13:

$$A_{ij}=\left\{\begin{array}{cc} 1, & \left(i,j\right)\in E_{\text{ppi}}\\ 0, & \text{otherwise} \end{array}\right.$$ (13)

Although $S\in R^{n\times n}$ can measure potential similarity between nodes, it may introduce false-positive links between unrelated nodes (Xiang et al., 2024). Therefore, an improved adjacency matrix $\hat{S}$ is constructed as shown in Equation 14:

$${\hat{S}}_{ij}=\left\{\begin{array}{cc} S_{ij}, & S_{ij}\geq \min\left\{S_{ik}|k\in N\left(v_{i}\right)\right\}\\ 0, & \text{otherwise} \end{array}\right.$$ (14)

where $N\left(v_{i}\right)$ denotes the neighborhood of node $v_{i}$ , and $\min\left\{S_{ik}|k\in N\left(v_{i}\right)\right\}$ represents the minimum similarity between node $v_{i}$ and all its neighbors. If $S_{ij}$ is greater than or equal to this threshold, the similarity is preserved; otherwise, it is removed.

Using this similarity graph, an enhanced adjacency matrix $\tilde{A}$ is constructed as expressed in Equation 15:

$${\tilde{A}}_{ij}=A_{ij}+\mu {\hat{S}}_{ij}$$ (15)

where $A_{ij}$ is the PPI adjacency value between nodes $v_{i}$ and $v_{j}$ , and μ is a balancing hyperparameter.

We perform graph convolution to obtain the first-layer node features $\varphi^{\left(1\right)}$ , which serve as the input to the first layer of hypergraph convolution. The computation of $\varphi^{\left(1\right)}$ is shown in Equation 16:

$$\varphi^{\left(1\right)}=\left(D^{-\frac{1}{2}}\tilde{A}D^{-\frac{1}{2}}\varphi^{\left(0\right)}W_{\text{ppi}}^{\left(0\right)}\right)$$ (16)

where D is the degree matrix of $\tilde{A}$ , $W_{\text{ppi}}^{\left(0\right)}$ is the node weight matrix of the PPI network, and $\varphi^{\left(0\right)}$ is the initial node adjacency vector in the PPI network.

A two-layer hypergraph convolutional network is constructed, using ReLU as activation. The first layer aggregates information from immediate hyperedge neighbors, while the second layer propagates information from more distant nodes, resulting in richer node embeddings. The two-layer hypergraph convolutions are defined in Equation 17 and Equation 18:

$$Z^{\left(1\right)}=ReLU\left(D_{v}^{-\frac{1}{2}}HWD_{e}^{-\frac{1}{2}}H^{T}D_{v}^{-\frac{1}{2}}\varphi^{\left(1\right)}\theta \right)$$ (17)

$$Z^{\left(2\right)}=ReLU\left(D_{v}^{-\frac{1}{2}}HWD_{e}^{-\frac{1}{2}}H^{T}D_{v}^{-\frac{1}{2}}Z^{\left(1\right)}\theta \right)$$ (18)

where θ is the learnable hypergraph convolution parameter matrix. The second hypergraph convolution $Z^{\left(2\right)}$ is the corresponding node embedding representation.

For each hypergraph in $G_{\text{all}}=\left\{G_{1},G_{2},\ldots ,G_{he}\right\}$ , we obtain a node embedding set $GZ_{\text{all}}=\left\{GZ^{1},GZ^{2},\ldots ,GZ^{\text{he}}\right\}.$ If node $v_{i}$ in the he -th hypergraph corresponds to a “supernode”, its embedding is redistributed back to its constituent nodes in the original PPI network. Suppose “supernode” n in the he -th hypergraph corresponds to node set SN(n) . Then the embedding of node $v_{i}$ is computed as in Equation 19:

$$DG_{\;c_{i}^{\text{he}}}^{\text{he}}\left(v_{i}\right)=\omega \left(v_{i}\right)\cdot GZ_{n}^{\text{he}}$$ (19)

where $\omega \left(v_{i}\right)$ is the normalized weight of node $v_{i}$ within its module.

Finally, the hypergraph node embedding for node $v_{i}$ is obtained by concatenating its embeddings from all hypergraph levels, as shown in Equation 20:

$$FD\left(v_{i}\right)=\left[DG_{\;c_{i}^{\text{he}}}^{1}\left(v_{i}\right),DG_{\;c_{i}^{\text{he}}}^{2}\left(v_{i}\right),\ldots ,DG_{\;c_{i}^{\text{he}}}^{\text{he}}\left(v_{i}\right)\right]$$ (20)

where $FD\left(v_{i}\right)$ is the final embedding of node $v_{i}$ , and $c_{i}^{he}$ denotes the hypergraph layer to which node $v_{i}$ belongs.

### 2.4 Cluster generation

In the clustering phase, a weighted PPI network $G_{w}$ is constructed by computing the cosine similarity between the protein embeddings derived from Equation 20. Let the embedding vector of protein node $v_{i}$ be $HD\left(v_{i}\right)=\left[v_{i1},v_{i2},\ldots ,v_{ig_{i}}\right]$ , and the embedding vector of node $v_{j}$ be $HD\left(v_{j}\right)=\left[v_{j1},v_{j2},\ldots ,v_{jg_{j}}\right]$ . Their cosine similarity is computed as shown in Equation 21:

$$\cos\left(v_{i},v_{j}\right)=\frac{HD\left(v_{i}\right)\cdot HD\left(v_{j}\right)}{\left\|HD\left(v_{i}\right)\|\|HD\left(v_{j}\right)\right\|}=\frac{\overset{g_{i}}{\underset{\ell =1}{\sum }}\left(v_{i\ell }\cdot v_{j\ell }\right)}{\sqrt{\overset{g_{i}}{\underset{\ell =1}{\sum }}v_{i\ell }^{2}}\;\sqrt{\overset{g_{j}}{\underset{\ell =1}{\sum }}v_{j\ell }^{2}}}.$$ (21)

A core–attachment strategy is then employed to identify protein complexes from the weighted PPI network $G_{w}$ . This strategy consists of two steps: (1) detecting core proteins and (2) attaching additional proteins to expand the cores into full complexes. A depth–first search is applied to enumerate all maximal cliques containing at least three protein nodes, forming the candidate core set CCSet . These cliques are sorted in descending order according to their density defined in Equation 22:

$$density\left({CC}_{k}\right)=\underset{v_{i},\;v_{j}\in {CC}_{k}}{\sum }\cos\left(v_{i},v_{j}\right),$$ (22)

where ${CC}_{k}\in CCSet$ denotes the k -th candidate core in the set $CCSet=\left\{{CC}_{1},{CC}_{2},\ldots ,{CC}_{k}\right\}$ .

Suitable seed cores SCSet are then selected from CCSet . First, the clique with the highest density, ${CC}_{1}$ , is added to SCSet . For each remaining clique ${CC}_{i}$ , overlapping proteins with ${CC}_{1}$ are removed. If the remaining part of ${CC}_{i}$ satisfies $|{CC}_{i}-{CC}_{1}|\geq 3$ , then ${CC}_{i}$ is updated as ${CC}_{i}-{CC}_{1}$ ; otherwise, it is removed from CCSet , since protein complexes with fewer than three proteins are usually considered biologically unreliable. This process is repeated until no cliques remain. The resulting seed core set is denoted as $SCSet=\left\{{SC}_{1},{SC}_{2},\ldots ,{SC}_{pc}\right\}$ .

Next, core members are added to each seed core by combining cluster density, neighborhood relationships, and the presence of triangles and squares in the PPI network. A core node $v_{i}$ is selected from SCSet , and its first-order neighbors are evaluated. Let ${NE}_{1}$ denote the first neighbor added to the core. If adding ${NE}_{1}$ increases the cluster density, then ${NE}_{1}$ becomes a core cluster member; otherwise, the remaining neighbors are evaluated. For each seed core ${SC}_{i}\in SCSet$ , if core cluster $Den\left({SC}_{i}\right)\geq dens$ , the seed core is regarded as a valid core cluster and included in CVSet , where dens is a parameter. The core cluster set CVSet is defined as $CVSet=\left\{{CV}_{1},{CV}_{2},\ldots ,{CV}_{\mathrm{pcp}}\right\}$ . The cluster density ${SC}_{i}$ is computed in Equation 23:

$$Den\left({SC}_{i}\right)=\left\{\begin{array}{cc} 0, & \text{if }N\left({SC}_{i}\right)<2,\\ \frac{2\times E\left({SC}_{i}\right)}{N\left({SC}_{i}\right)\times \left(N\left({SC}_{i}\right)-1\right)}, & \text{otherwise} \end{array}\right.$$ (23)

where $E\left({SC}_{i}\right)$ and $N\left({SC}_{i}\right)$ denote the numbers of edges and nodes in cluster ${SC}_{i}$ , respectively.

To ensure that the expanded core cluster maintains strong internal connectivity, we select appropriate accessory protein nodes to add to the core clusters. Considering the core clusters as hyperedges (seeds), new protein nodes are gradually adsorbed to expand these initial core clusters, thus obtaining the complete protein complex. In order to ensure that the hyperedge still has high internal connectivity after the candidate protein nodes are added, this paper introduces the HyperedgeDensity in Equation 24:

$$HyperedgeDensity\left({CV}_{i}\cup \left\{v_{g}\right\}\right)=\frac{2\times \underset{e\in \left({CV}_{i}\cup \left\{v_{g}\right\}\right),\;|e|=2}{\sum }\gamma \left(e\right)}{|{CV}_{i}\cup \left\{v_{g}\right\}|\cdot \left(|{CV}_{i}\cup \left\{v_{g}\right\}|-1\right)}$$ (24)

where γ(e)=1 if protein pair e has an edge in the original PPI network; otherwise, γ(e)=0 .

In addition, relying on the Hyperedge Density alone for adsorption may lead to excessive node sharing between different cores, resulting in functional confusion and blurred boundaries between different protein complexes. To avoid this situation, this paper introduces Hyperedge Overlap to clarify nodes’ difference between core clusters, defined in Equation 25, to distinguish differences between core clusters:

$$HyperedgeOverlap=\underset{j\neq i}{\max}\frac{\left|\left({CV}_{i}\cup \left\{v_{g}\right\}\right)\cap {CV}_{j}\right|}{\left|\left({CV}_{i}\cup \left\{v_{g}\right\}\right)\cup {CV}_{j}\right|}$$ (25)

A candidate protein node $v_{g}$ that has not yet been assigned to any core cluster is added to core cluster $CV_{i}$ when HyperedgeDensity ≥ε and HyperedgeOverlap ≤θ are both satisfied.

### 2.5 Time complexity analysis

The overall computational complexity comprises four main components: hypergraph construction, hierarchical compression, node embedding, and cluster generation. Let n and m denote the number of nodes and edges in the PPI network. L denotes the number of hierarchical compression levels, while d denotes the embedding dimension.

Specifically, in the hypergraph construction phase, the PPI network is transformed into a hypergraph by generating hyperedges and building a node-hyperedge incidence matrix. This step has a time complexity of O(m) . In the hierarchical compression stage, hypergraph modules are optimized and aggregated. Each layer of the hypergraph is partitioned into communities, and each community is merged into a “supernode”. If the number of edges in layer i is $m_{i}$ , the time complexity of this process is $\sum_{i=0}^{L-1}O\left(m_{i}\right)$ . In the node embedding stage, two hypergraph convolutions are applied to each hypergraph layer. The time complexity of each convolution is $O\left(m_{i}\cdot d\right)$ . Therefore, the total time complexity of this stage is $\sum_{i=0}^{L-1}O\left(m_{i}\cdot d\right)$ . In the node clustering stage, the algorithm performs maximal clique enumeration within the local neighborhood of each node. It is well known that the number of maximal cliques in an arbitrary graph can reach $3^{n}$ in the worst case (Moon-Moser theorem), and therefore maximal clique enumeration is NP-hard in general. However, our algorithm does not enumerate maximal cliques over the entire PPI network. Instead, the enumeration is restricted to the local neighborhood of each node, whose size is approximately equal to the average degree k . Biological networks, including PPI networks, are typically sparse and exhibit heavy-tailed degree distributions, which significantly limits the size of local neighborhoods and prevents worst-case exponential behavior from occurring in practice. Let f(k) denote the actual number of maximal cliques in a neighborhood of size k . Although f(k) is theoretically upper-bounded by $3^{k}$ , empirical observations on biological networks show that f(k) grows slowly and remains close to a low-degree polynomial in k . Therefore, the practical complexity of one clique enumeration step can be expressed as O(f(k)) , and the total cost across all nodes becomes O(n⋅f(k)) . Since in real PPI networks k≪n and f(k) is small due to network sparsity, the effective runtime of this stage is well approximated by $O\left(nk^{2}\right)$ . In practice, HLCA has been successfully applied to larger datasets such as BIOGRID and DIP, demonstrating its scalability and confirming that the algorithm can handle larger networks effectively. Consequently, the overall computational complexity of the HLCA framework remains within the $O\left(nk^{2}\right)$ scale.

### 2.6 Evaluation metrics

To evaluate the performance of the proposed HLCA algorithm, we use several metrics, including F-measure, precision, recall, and Accuracy (ACC). The F-measure is calculated based on the precision and recall of the test. Precision represents the ratio of true positives to all positive results, including false positives, while recall represents the ratio of true positives to all actual positive samples. ACC is the geometric mean of Sensitivity (Sn) and Positive Predictive Value (PPV).

To compare the detected protein complexes with known protein complexes, the neighborhood affinity score $OS\left(pc_{i},gc_{j}\right)$ between them is defined as Equation 26:

$$OS\left(pc_{i},gc_{j}\right)=\frac{{\left|pc_{i}\cap gc_{j}\right|}^{2}}{\left|pc_{i}\right|\times \left|gc_{j}\right|}$$ (26)

where $pc_{i}$ denotes a identified protein complex and $gc_{j}$ denotes a known (ground truth) complex. The neighbourhood affinity score OS measures similarity between two complexes, with higher OS values indicating greater similarity. This metric also facilitates the overlap assessment between the detected complex $pc_{i}$ and the standard complex $gc_{j}$ . If $OS\left(pc_{i},gc_{j}\right)$ is greater than or equal to a predefined threshold δ , $pc_{i}$ and $gc_{j}$ are considered a match.

The precision, recall, and F-measure metrics are calculated using Equations 27–29:

$$Precision=\frac{\left|\left\{\;pc_{i}\in pc|\exists \;gc_{j}\in gc,\;OS\left(pc_{i},gc_{j}\right)\geq \delta \;\right\}\right|}{\left|pc\right|}$$ (27)

$$Recall=\frac{\left|\left\{\;gc_{j}\in gc|\exists \;pc_{i}\in pc,\;OS\left(pc_{i},gc_{j}\right)\geq \delta \;\right\}\right|}{\left|gc\right|}$$ (28)

$$F-\text{measure}=\frac{2\times Precision\times Recall}{Precision+Recall}$$ (29)

In addition, Accuracy (ACC), sensitivity (Sn), and positive predictive value (PPV) are defined as shown in Equations 30–32:

$$Sn=\frac{\overset{\left|gc\right|}{\underset{i=1}{\sum }}\underset{1\leq j\leq |pc|}{\max}T_{ij}}{\overset{\left|gc\right|}{\underset{i=1}{\sum }}N_{i}}$$ (30)

$$PPV=\frac{\overset{\left|gc\right|}{\underset{i=1}{\sum }}\underset{1\leq j\leq |pc|}{\max}T_{ij}}{\overset{\left|pc\right|}{\underset{j=1}{\sum }}\overset{\left|gc\right|}{\underset{i=1}{\sum }}T_{ij}}$$ (31)

$$ACC=\sqrt{Sn\times PPV}$$ (32)

Here, $T_{ij}$ denotes the number of proteins shared between the known protein complex i and the detected protein complex j , and $N_{i}$ denotes the number of proteins in the known complex i .

F-measure and Accuracy can each reflect the algorithm’s performance in different dimensions, but the single use of one of the metrics may ignore the comprehensiveness of the algorithm’s performance. According to previous studies (Vlasblom and Wodak, 2009; Altaf-Ul-Amin et al., 2006; Omranian et al., 2021), F-measure reflects the algorithm’s comprehensive balance between the precision and recall of the prediction results. In contrast, Accuracy can intuitively reflect the overall agreement between the algorithm’s prediction results and the real complexes. Combining these two metrics helps to assess the algorithm’s performance more objectively and comprehensively. In this paper, we use F-measure + Accuracy as a comprehensive evaluation index, denoted as F1 + ACC.

## 3 Result

### 3.1 Experimental datasets

The PPI networks used in this paper are Gavin1 (Gavin et al., 2002; Wang et al., 2025a), Gavin2 (Gavin et al., 2006; Abbas et al., 2025), K_extend (Krogan et al., 2006; Wang et al., 2025b), DIP (Xenarios et al., 2002; Wang et al., 2025b), and BioGRID (Oughtred et al., 2019; Papik et al., 2025). As a complete species-specific set of PPI networks, these yeast datasets are most widely used in protein complex identification studies. Therefore, yeast is selected as the experimental object. CYC2008 (Pu et al., 2009) and MIPS (Brohee and Van Helden, 2006) are adopted as the ground truth for yeast proteins to conduct parameter analysis and evaluate the clustering results. Table 1 provides detailed information about these PPI networks. The datasets are then preprocessed to remove all self-interactions and duplicate interactions.

**TABLE 1:** PPI datasets.

Dataset	Gavin1	Gavin2	K_extend	DIP	BioGRID
Proteins	1,352	1,430	3,672	4,930	4,187
Interactions	3,210	6,531	14,317	17,201	20,454

### 3.2 Parameter analysis

To study the influence of key parameters on model performance, a comprehensive sensitivity analysis is conducted for the three main parameters-cluster density threshold dens∈[0.1,1] , hyperedge density threshold ε∈[0.1,1] , and hyperedge overlap threshold θ∈[0.1,1] , used in the proposed algorithm. These parameters play critical roles in determining the structure and quality of the predicted protein complexes.

**FIGURE 2:**
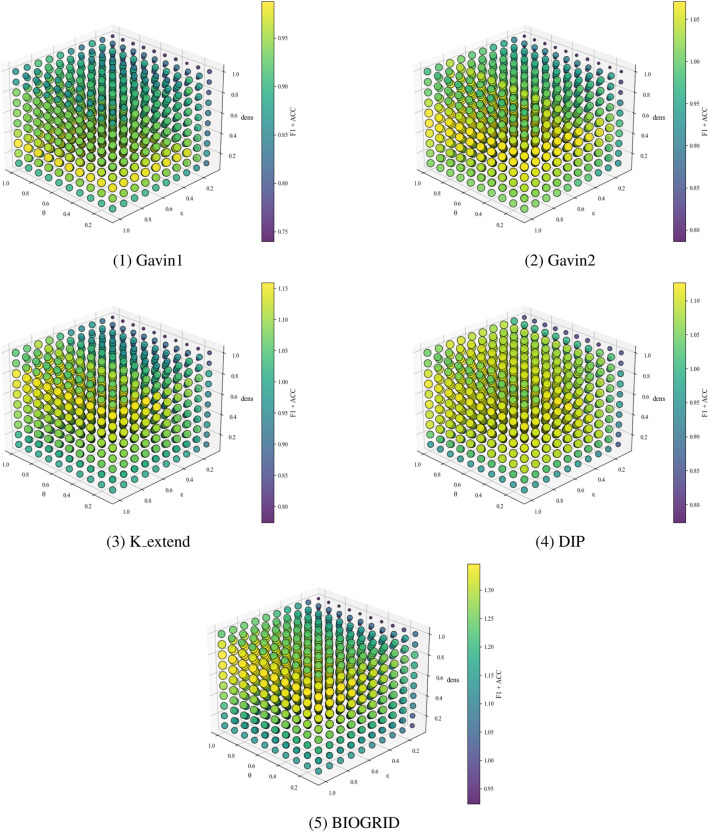
CYC2008 as benchmarks, parametric analysis on different datasets.

**FIGURE 3:**
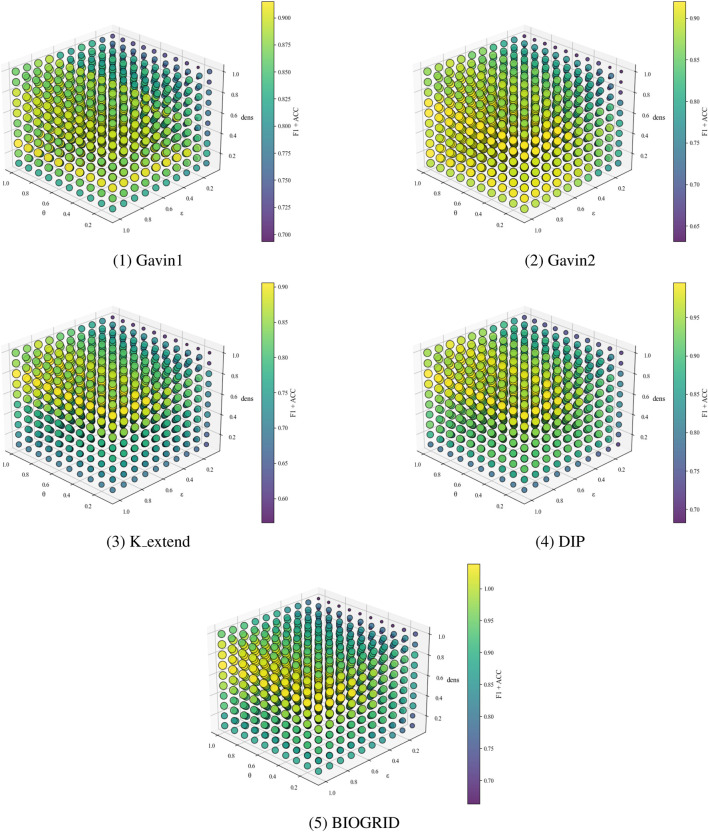
MIPS as benchmarks, parametric analysis on different datasets.

A total of 1,000 parameter configurations are evaluated by performing a grid search across the three parameters dens , ε , and θ . For each configuration, experiments are carried out on the Gavin1, Gavin2, K_extend, DIP, and BIOGRID datasets, using CYC2008 and MIPS as the ground truths. The sensitivity analysis results are presented in Figures 2, 3.

Experimental results show that increasing the cluster density threshold dens leads to a rapid improvement in performance, followed by stabilization across all datasets. The overall F1 + ACC metric peaks when dens is set around 0.8, indicating that this value allows the model to effectively capture compact and robust core protein complexes. Under this setting, the model can effectively capture more reasonable and robust core clusters. The hyperedge density threshold ε also significantly impacts performance across datasets. While variation is observed among datasets, performance tends to be optimal when ε reaches 0.7, where F1 + ACC consistently remains high. This suggests that higher ε values facilitate the inclusion of informative attachment nodes, thereby enhancing protein complex identification accuracy. The hyperedge overlap threshold θ exhibits notable sensitivity as well. Performance degrades when θ is set too low or too high. This situation indicates that extreme values may either underrepresent or overrepresent functional modules. The optimal performance and stability are observed when θ is set to 0.5, which balances module distinctiveness and inter-complex sharing.

Although different parameter settings may yield slightly better results on specific datasets, the parameter combination of dens=0.8 , ε=0.7 , and θ=0.5 consistently outperforms baseline algorithms across all evaluation metrics. While tuning parameters individually per dataset could further enhance performance, it may compromise the model’s generalizability. Overall, when dens∈[0.7,0.9] , ε∈[0.6,0.8] , and θ∈[0.4,0.6] , the algorithm maintains high F1 + ACC performance across different datasets. This indicates that parameter values within these ranges deliver robust and reliable performance. Considering overall performance, stability, and cross-dataset generalization, the parameter combination is selected as the final configuration. This selection ensures strong performance across diverse datasets and robust structural consistency, highlighting the effectiveness and rationale behind the parameter design. This parameter combination ensures that HLCA performs reliably across different datasets without the need for dataset-specific parameter tuning, addressing concerns related to the sensitivity and reproducibility of results.

### 3.3 Effectiveness analysis of hypergraph learning and improved core-attachment strategy

**FIGURE 4:**
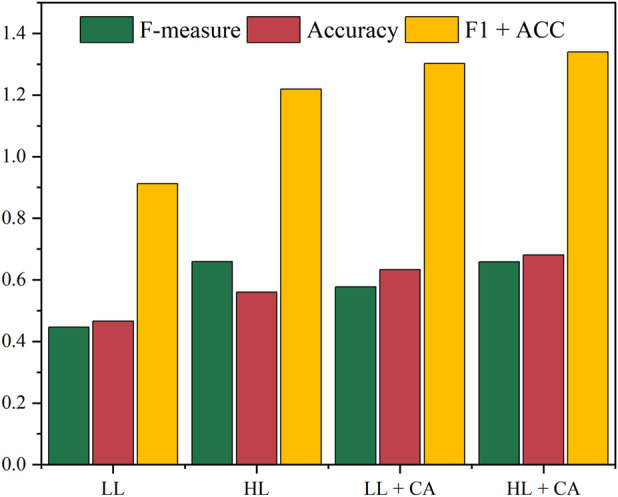
CYC2008 as ground truth, effectiveness analysis of hypergraph learning and improved core-attachment strategy on BIOGRID dataset (LL, low-order topology; HL, high-order topology; LL + CA, low-order topology with improved core-attachment strategy; HL + CA, high-order topology with improved core-attachment strategy).

In this study, the HLCA algorithm integrates hypergraph learning with an improved core-attachment strategy for protein complex identification. In the hypergraph learning component, higher-order topological information is incorporated into the PPI network. This information enables a more effective modeling of multi-protein cooperative patterns and improves the representation of complex interaction relationships. In the core-attachment component, tightly associated protein nodes are grouped into cores based on cluster density. Attachment proteins are then assigned according to node metrics and higher-order topological structure. To evaluate the effect of higher-order topology and the improved strategy on recognition performance, a series of comparative experiments is designed and conducted. The results of the experiments are shown in Figure 4 (with CYC2008 as the ground truth and the BIOGRID dataset as an example). As shown in Figure 4, LL denotes the use of only low-order topological information, and HL denotes the use of only high-order topological information. LL + CA represents the use of low-order topological information combined with the improved core-attachment strategy, while HL + CA represents the use of high-order topological information combined with the improved core-attachment strategy.

This paper first compares the effects of low-order and high-order topological information on protein complex identification. In this setting, the traditional core-attachment strategy is used to identify protein complexes. The experimental results show that higher-order topology information is superior to lower-order topology information, with F-measure and Accuracy improved by about 47.89% and 20.10%, respectively. This advantage arises from the ability of higher-order topological information to model many-to-many synergistic relationships among proteins, beyond simple binary interactions in PPI networks. As a result, it can effectively capture complex interactions and modular structures that are often difficult to detect in a conventional PPI network.

Further, this paper analyzes the impact of the proposed improved core-attachment strategy on protein complex recognition performance. Based on low-order and higher-order topological information, this paper introduces node metrics in the core search and attachment addition process. The experimental results demonstrate that the proposed improved core-attachment strategy consistently outperforms the original version, regardless of the topological order. When combined with low-order topology, the improved strategy increases F-measure and Accuracy by 13.11% and 16.72%, respectively. Further improvements are observed under higher-order topology, where Accuracy increases by an additional 12.1% over the original strategy. Moreover, compared to low-order topology, the incorporation of higher-order topology further enhances F-measure and Accuracy by 8.16% and 4.75%, respectively, when applying the improved strategy. In summary, the proposed algorithm integrates high-order topological information with node metrics to achieve superior performance in both F-measure and Accuracy. These results suggest that incorporating nodal metrics enhances the ability to capture protein complexes’ structural characteristics, thereby improving recognition effectiveness and predictive accuracy.

### 3.4 Comparative analysis of hypergraph network embedding experiments

**FIGURE 5:**
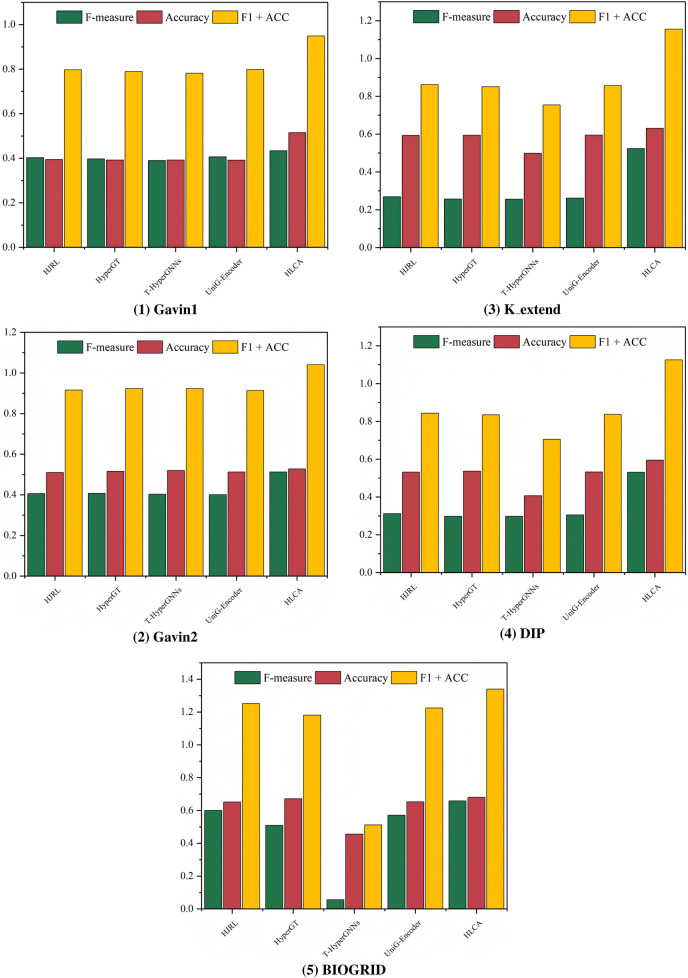
CYC2008 as ground truth, evaluation results by different hypergraph neural network algorithms.

**FIGURE 6:**
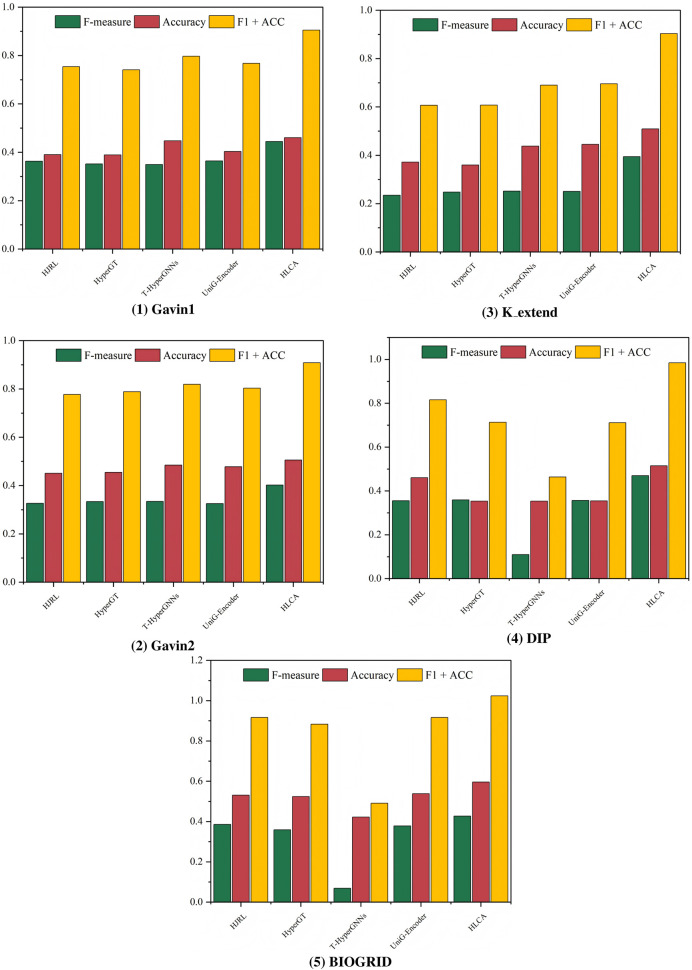
MIPS as ground truth, evaluation results by different hypergraph neural network algorithms.

Building upon the incorporation of high-order hypergraph topology, the HLCA algorithm further incorporates a hypergraph network embedding framework and integrates a hierarchical compression strategy into the embedding process. To evaluate the effectiveness of this integration for protein complex identification, a series of comparative experiments were conducted against representative hypergraph neural network methods, including HJRL (Yan et al., 2024), HyperGT (Liu et al., 2024), UniG-Encoder (Zou et al., 2024), and T-HyperGNNs (Wang et al., 2024). The four hypergraph neural network models were used solely to generate their respective node embeddings. These embeddings were then employed for subsequent protein complex identification. The experimental results are shown in Figures 5, 6. In these figures, the horizontal axis denotes the compared algorithms, and the vertical axis denotes the corresponding evaluation metrics.

HJRL adopts a cross-expansion strategy. In this strategy, hypernodes and hyperedges in the original hypergraph are mapped to nodes in an expanded graph. Additional connecting edges are introduced to model their interrelationships. This strategy enables joint learning of hypernodes and hyperedges representations while maintaining a unified graph structure. However, this conversion increases the graph size and computational complexity. It also leads to redundancy and information interference, reducing embedding compactness. In contrast, the HLCA algorithm employs a hierarchical compression strategy, dividing the original hypergraph into multiple compact smaller hypergraphs. Hypergraph convolution is applied within each smaller hypergraph to learn embedding representations, which are subsequently merged to form comprehensive node representations. This design effectively reduces computational overhead, avoids redundancy from graph expansion, and better preserves local structural information. Experimental results on the Gavin1, Gavin2, K_extend, DIP, and BIOGRID datasets, using CYC2008 as the ground truth, demonstrate that HLCA consistently outperforms HJRL in F-measure and Accuracy. Specifically, it achieves a 21.43% improvement in F1 + ACC. Similar results are observed under the MIPS ground truth, with a 23.61% improvement in F1 + ACC. These results validate the practical advantages of the hierarchical compression strategy in modeling complex hypergraph structures.

HyperGT is a Transformer-based hypergraph neural network that leverages self-attention mechanisms within hyperedges to model local dependencies and learn expressive node representations. It represents a typical local modeling strategy in hypergraph learning. While HyperGT and HLCA emphasize local structural modeling, HLCA introduces hypergraph modularity that divides the hypergraph into smaller hypergraphs. This allows for more comprehensive learning of localized information. Consequently, the resulting embeddings are more compact and expressive. Experimental comparisons on the same five datasets with CYC2008 and MIPS ground truth show that HLCA achieves superior F-measure and Accuracy scores on most datasets. The F1 + ACC improves 23.42% and 27.55%, respectively. These results highlight the importance of structured local representation in protein complex identification tasks.

UniG-Encoder is a global hypergraph self-encoder that learns representations by reconstructing the entire hypergraph structure. It captures global dependencies through an encode–decode process to preserve hyperedge reconstruction fidelity. The HLCA algorithm also incorporates global information through a hierarchical compression strategy that preserves topological structure. Node representations from multiple levels are merged to synthesize features from different topological perspectives, capturing both macro structure and fine-grained connectivity. This approach mitigates the risk of over-smoothing in global encoders and better preserves key local variations. Experimental results demonstrate that HLCA outperforms UniG-Encoder across all datasets under CYC2008 and MIPS ground truth. The F1 + ACC gains of 22.66% and 22.57%, respectively, underscore the effectiveness of the proposed method in modeling global structures while retaining critical local features.

T-HyperGNNs is designed to integrate both local and global information in hypergraph learning. It applies T-spectral convolution to extract higher-order global structural patterns and combines this with T-space convolution to model local dependencies. This multi-level modeling aims to capture comprehensive topological insights. Similarly, HLCA incorporates both local and global structural modeling through hierarchical compression. The hypergraph is divided into smaller hypergraphs for localized convolutional learning, where the embeddings are aggregated to form comprehensive node representations. This hierarchical compression strategy facilitates effective local and global topological information integration. Experimental evaluations confirm that HLCA consistently outperforms T-HyperGNNs across all datasets. Under the CYC2008 ground truth, the F1 + ACC metric improves by 38.7%. Under the MIPS ground truth, the improvement reaches 29.3%. These results demonstrate the superior performance of the proposed method in both local–global integration and representation effectiveness.

The HLCA algorithm demonstrates consistently superior performance in protein complex identification tasks in the above comparison of hypergraph neural network experiments. The hierarchical compression strategy not only reduces computational overhead but also enhances both local and global feature learning. Improvements in F1 + ACC ranging from 21.43% to 38.7% across multiple datasets and baselines demonstrate the model’s strong empirical performance. These results highlight its robustness, expressive power, and suitability for modeling complex biological networks.

### 3.5 Comparative analysis of protein complex identification experiments

To comprehensively evaluate the performance of the HLCA algorithm, a comparison is conducted against a range of commonly used protein complex identification algorithms, including the complete enumeration algorithm CFinder (Adamcsek et al., 2006), heuristic-based algorithms DPClus (Altaf-Ul-Amin et al., 2006), IPCA (Li et al., 2008a) and SEGC (Wang et al., 2017), the core-attachment algorithms CORE (Leung et al., 2009) and COACH (Wu et al., 2009), the uncertain graph model DCU (Zhao et al., 2014), the annotation-driven core-attachment algorithm WCOACH (Kouhsar et al., 2015), the flow-based algorithm SR-MCL (Shih and Parthasarathy, 2012), the graph partitioning algorithm BOPS (Wang et al., 2025a), the heuristic-based algorithms GCAPL (Wang et al., 2023) and DMPC (Sahoo et al., 2023), and the graph embedding method DPCMNE (Meng et al., 2021).

**FIGURE 7:**
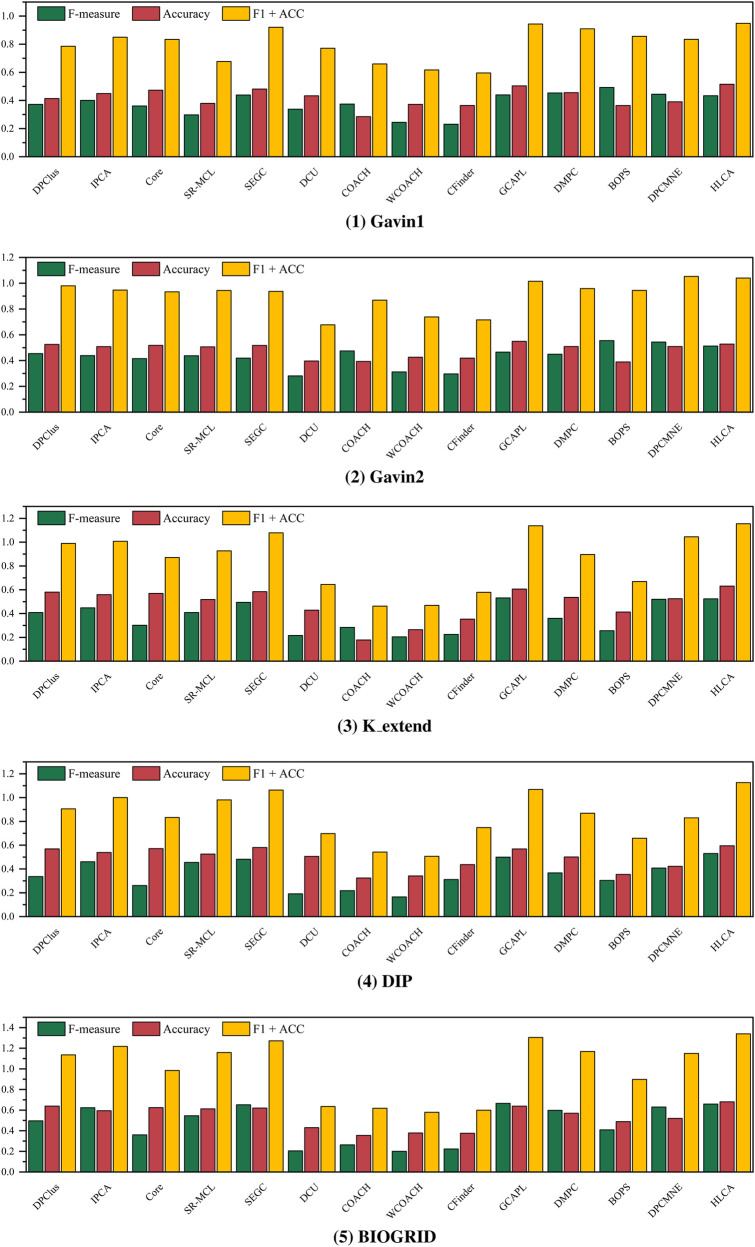
CYC2008 as ground truth, performance comparison of HLCA with baseline algorithms on different datasets.

Figure 7 summarizes the performance of the HLCA algorithm on the Gavin1, Gavin2, K_extend, DIP, and BIOGRID datasets, using CYC2008 as the ground truth, where the horizontal axis denotes the compared algorithms and the vertical axis denotes the corresponding metric values (F-measure, Accuracy, and F1+ACC). The HLCA algorithm achieves competitive or superior performance compared with most baseline methods in terms of F-measure and Accuracy across datasets. Notably, the F1+ACC values of HLCA are higher than those of most baseline methods on these datasets. The improvements in the F1+ACC metric reach 15.99%, 13.94%, 32.58%, 30.25%, and 36.11% on Gavin1, Gavin2, K_extend, DIP, and BIOGRID, respectively. Performance gains are particularly evident on large-scale datasets such as K_extend, DIP, and BIOGRID. On these datasets, F-measure improves by 16.53%, 18.73%, and 20.71%, while Accuracy increases by 16.06%, 11.52%, and 15.40% on the same datasets.

**FIGURE 8:**
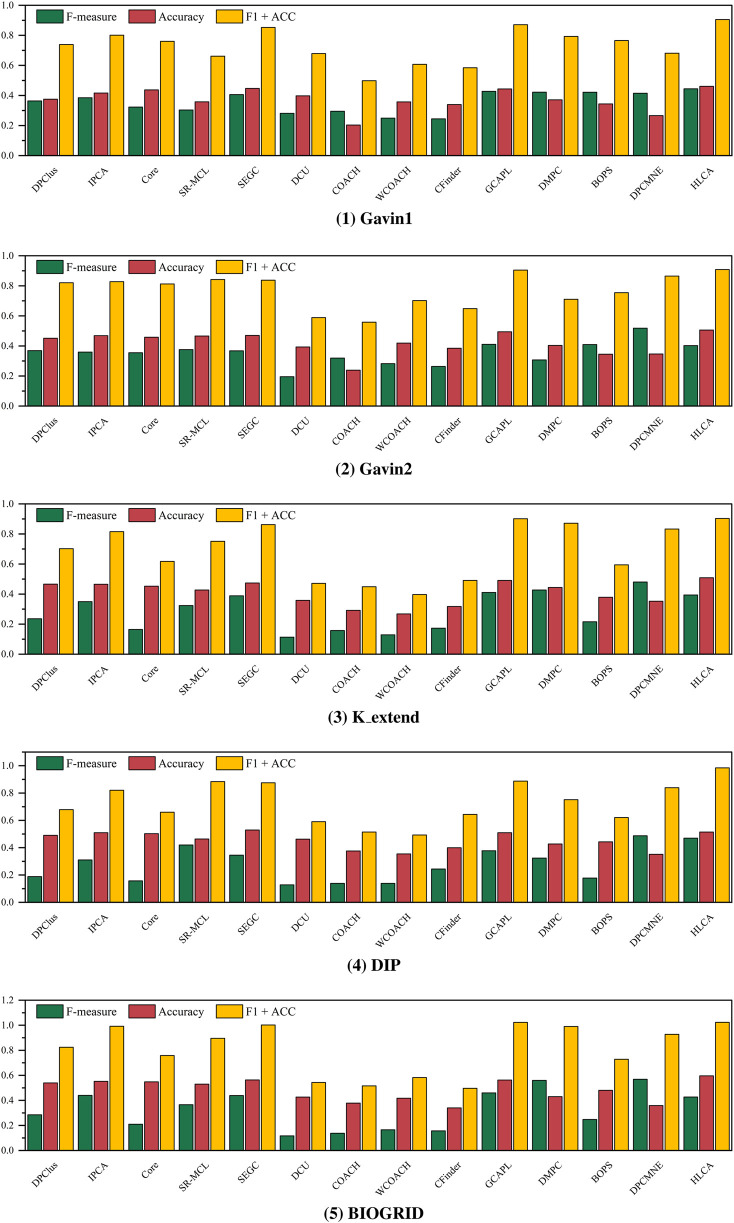
MIPS as ground truth, performance comparison of HLCA with baseline algorithms on different datasets.

Figure 8 presents the performance of the HLCA algorithm on the Gavin1, Gavin2, K_extend, DIP, and BIOGRID datasets, using MIPS as the ground truth, where the horizontal axis denotes the compared algorithms and the vertical axis denotes the corresponding metric values (F-measure, Accuracy, and F1+ACC). The HLCA algorithm achieves comparable or superior performance in terms of F-measure and Accuracy on most datasets. Specifically, improvements in F1+ACC over competing methods are observed as 19.02%, 14.89%, 22.95%, 27.23%, and 23.27% on Gavin1, Gavin2, K_extend, DIP, and BIOGRID, respectively.

To further verify the statistical significance of the results, Friedman and Nemenyi tests are conducted to evaluate the overall performance differences among the algorithms. For each evaluation metric on each dataset (F-measure, Accuracy, and F1 + ACC), algorithms are ranked separately, and the statistical significance of the observed differences is assessed using the Friedman test.

Using CYC2008 as the ground truth, the Friedman test is applied to assess average rankings across datasets under the three evaluation metrics. For F-measure, the average rankings are: 7.4 (DPClus), 5.8 (IPCA), 10.2 (CORE), 8 (SR-MCL), 5 (SEGC), 12.8 (DCU), 9 (COACH), 13.4 (WCOACH), 12 (CFinder), 2.6 (GCAPL), 6 (DMPC), 6.4 (BOPS), 3.6 (DPCMNE), and 2.8 (HLCA), with $P=7.431\times 10^{-6}$ . For Accuracy, the rankings are: 4.6 (DPClus), 6.2 (IPCA), 4.4 (CORE), 8.2 (SR-MCL), 3.6 (SEGC), 9.6 (DCU), 13.4 (COACH), 11.8 (WCOACH), 11.6 (CFinder), 2.6 (GCAPL), 7.4 (DMPC), 11.2 (BOPS), 9.2 (DPCMNE), and 1.2 (HLCA), with $P=5.464\times 10^{-7}$ . For F1 + ACC, the average rankings are: 6.6 (DPClus), 5 (IPCA), 8.8 (CORE), 7.4 (SR-MCL), 4.2 (SEGC), 11.4 (DCU), 12.4 (COACH), 13.2 (WCOACH), 12 (CFinder), 2.2 (GCAPL), 5 (DMPC), 8.8 (BOPS), 5.6 (DPCMNE), and 1.2 (HLCA), with $P=4.62\times 10^{-7}$ . These results indicate statistically significant differences among the algorithms, with the HLCA algorithm consistently achieving one of the highest rankings across all evaluation metrics.

Using MIPS as the ground truth, the Friedman test is applied to assess average rankings across all datasets for the three evaluation metrics. For F-measure, the average rankings are: 7.8 (DPClus), 6.4 (IPCA), 10 (CORE), 6.4 (SR-MCL), 5.6 (SEGC), 13.6 (DCU), 11.6 (COACH), 12.4 (WCOACH), 11.4 (CFinder), 2.8 (GCAPL), 4.8 (DMPC), 7 (BOPS), 1.8 (DPCMNE), and 3.4 (HLCA), with $P=1.009\times 10^{-6}$ . For Accuracy, the rankings are: 6 (DPClus), 4.2 (IPCA), 5.2 (CORE), 7.2 (SR-MCL), 2.2 (SEGC), 8.8 (DCU), 13 (COACH), 11.2 (WCOACH), 12 (CFinder), 2.8 (GCAPL), 8.6 (DMPC), 10 (BOPS), 12.6 (DPCMNE), and 1.2 (HLCA), with $P=6.852\times 10^{-8}$ . For F1 + ACC, the rankings are: 7.8 (DPClus), 5.2 (IPCA), 8.4 (CORE), 6.4 (SR-MCL), 3.8 (SEGC), 11.8 (DCU), 13.4 (COACH), 12.4 (WCOACH), 12 (CFinder), 2 (GCAPL), 6 (DMPC), 9.2 (BOPS), 5.6 (DPCMNE), and 1 (HLCA), with $P=1.713\times 10^{-7}$ . The results demonstrate statistically significant differences among the algorithms, with the HLCA algorithm consistently ranking among the top performers across all evaluation metrics.

**FIGURE 9:**
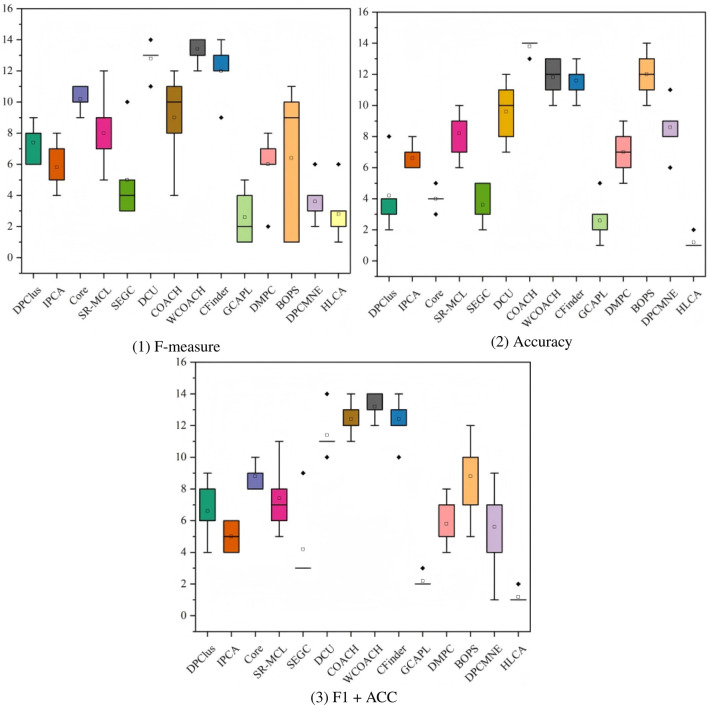
CYC2008 as ground truth, box plots of the rankings for the three metrics across different datasets.

Figure 9 displays the ranking box plots across all datasets of algorithm rankings for three evaluation metrics (F-measure, Accuracy, and F1 + ACC), using CYC2008 as the ground truth. The horizontal axis shows algorithm names, while the vertical axis represents rank values. Each box indicates the interquartile range, with the median marked by a horizontal line. Whiskers extend to the minimum and maximum non-outlier ranks, and outliers are displayed as individual points. This plot allows direct comparison of overall ranking performance and stability across datasets for different algorithms. The HLCA algorithm consistently ranks among the top-performing methods across all metrics, with particularly strong and stable Accuracy and F1 + ACC performance. In contrast, methods such as COACH and WCOACH exhibit higher rank variability. The presence of more fluctuations and outliers further indicates their lower stability. The P -values obtained from the Friedman tests (F-measure: $P=7.431\times 10^{-6}$ , Accuracy: $P=5.464\times 10^{-7}$ , F1 + ACC: $P=4.62\times 10^{-7}$ ) confirm that the observed performance differences among algorithms are statistically significant. These results highlight the HLCA algorithm’s advantage in accuracy and robustness.

**FIGURE 10:**
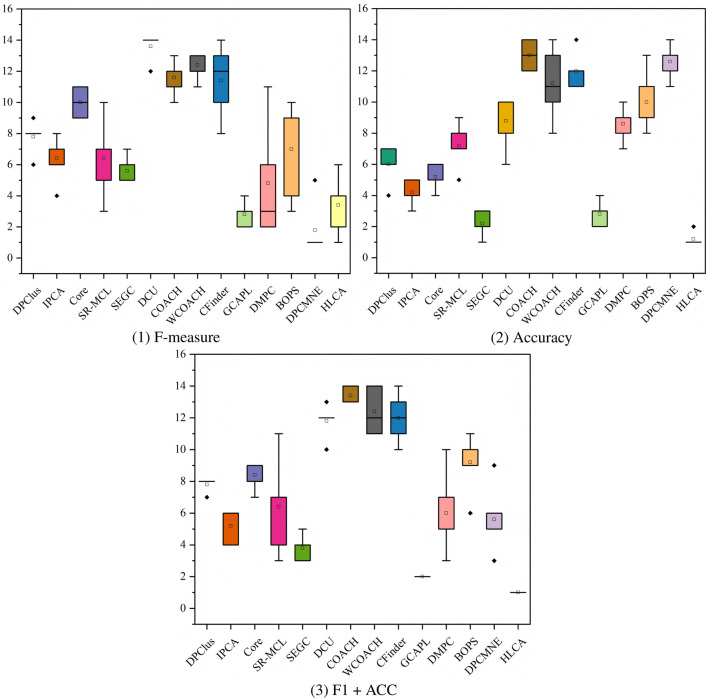
MIPS as ground truth, box plots of the rankings for the three metrics across different datasets.

Figure 10 presents the ranking box plots across all datasets of algorithm rankings for three evaluation metrics (F-measure, Accuracy, and F1 + ACC), using MIPS as the ground truth. The HLCA algorithm consistently achieves the highest rank across all metrics. It also exhibits the smallest interquartile range (IQR) with minimal deviation, indicating superior performance and high stability across datasets. In contrast, algorithms such as SR-MCL and DMPC show moderate performance on certain metrics but demonstrate greater rank variability. The Friedman test yields highly significant P -values (F-measure: $P=1.009\times 10^{-6}$ , Accuracy: $P=6.852\times 10^{-8}$ , and F1 + ACC: $P=1.713\times 10^{-7}$ ), confirming statistically significant differences among the algorithms.

The Nemenyi test is performed to assess the statistical significance of the observed ranking differences. The results confirm significant pairwise performance differences among the algorithms. The formula for computing the critical difference (CD) between methods is provided in Equation 33:

$$CD=q_{\alpha }\sqrt{\frac{a\left(a+1\right)}{6s}},$$ (33)

where a is the number of algorithms and s is the number of datasets. Based on the definition $q_{\alpha }=2.978$ given in (Zhao et al., 2018), for α=0.1 , the critical difference (CD) is 7.88 in this paper.

**FIGURE 11:**
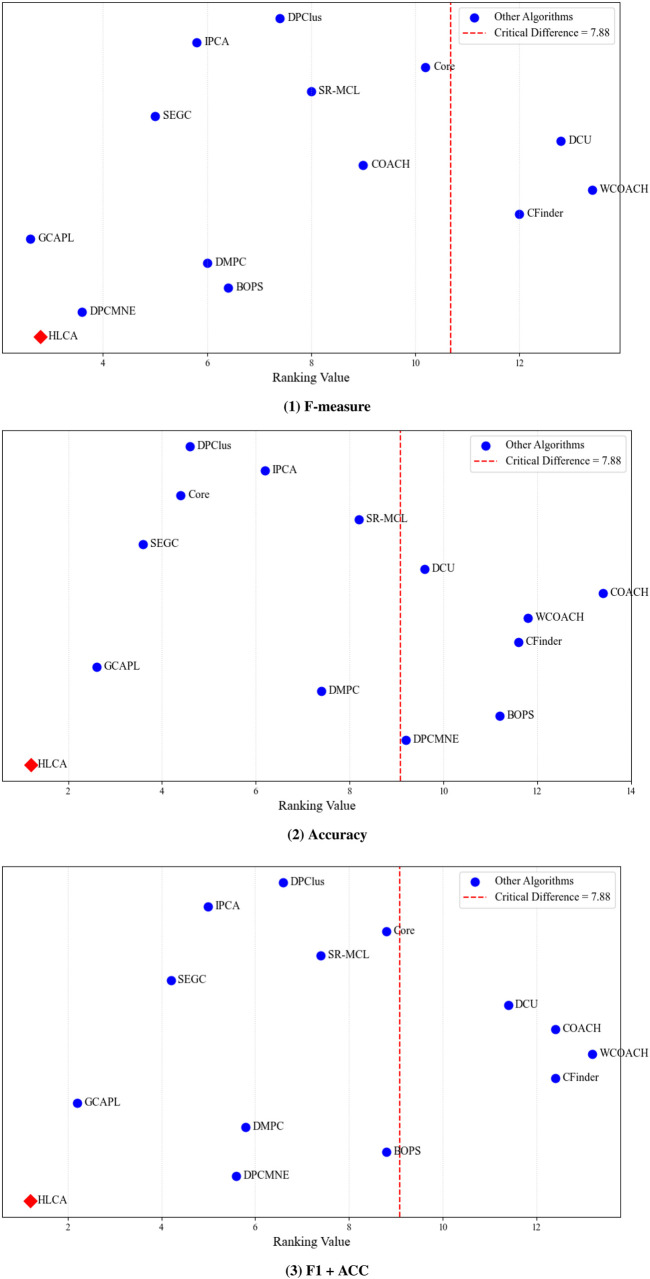
CYC2008 as ground truth shows the Nemenyi tests for the three metrics across different datasets.

**FIGURE 12:**
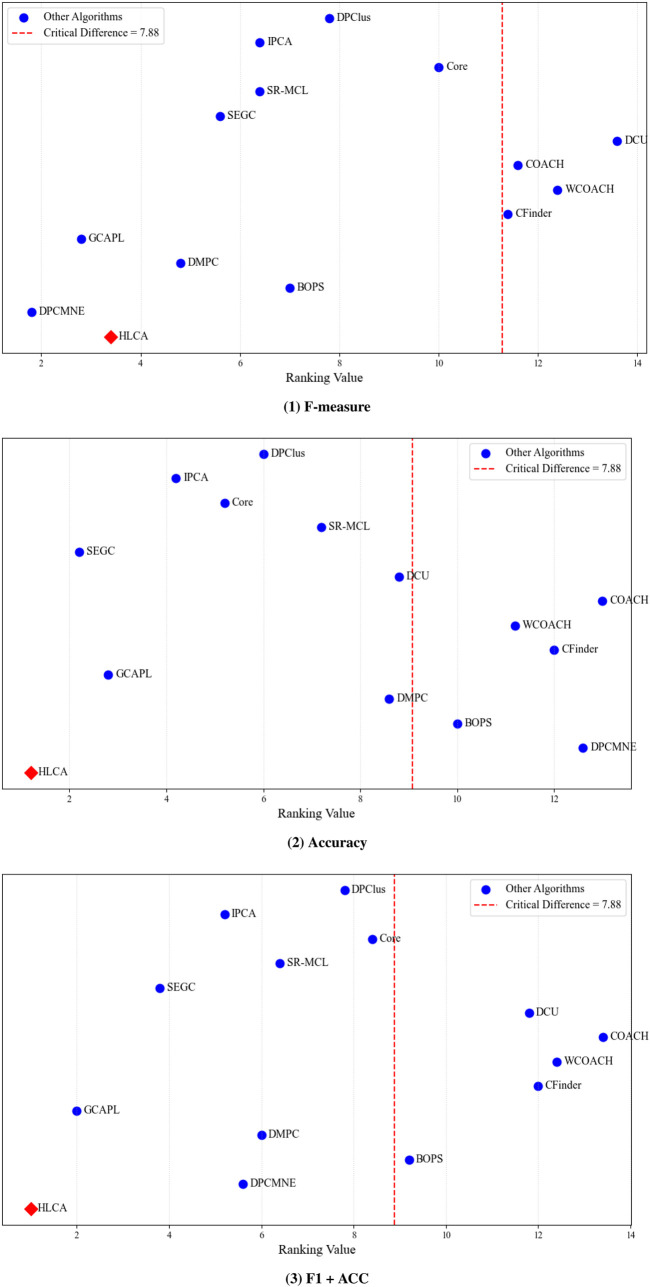
MIPS as ground truth shows the Nemenyi tests for the three metrics across different datasets.

The HLCA algorithm is evaluated using the Nemenyi test with CYC2008 and MIPS as ground truth. Results for the three performance metrics (F-measure, Accuracy, and F1 + ACC) are presented in Figures 11, 12. The average rank of each comparison algorithm is shown as a blue circle, and the HLCA algorithm is shown as a red diamond. When the horizontal distance between a red diamond and a blue circle is greater than or equal to the critical difference, the HLCA algorithm demonstrates significantly different performance from the corresponding comparison algorithm. Significant differences are indicated to the right of the red dashed line. On the CYC2008 ground truth, the HLCA algorithm achieves average rankings of 2.8, 1.2, and 1.2 for F-measure, Accuracy, and F1 + ACC, respectively. These results consistently place it among the top-performing methods. Notably, it ranks highest in Accuracy and F1 + ACC. Compared with other algorithms, the HLCA algorithm demonstrates superior overall performance. Moreover, the ranking gaps with most competing methods exceed the critical difference (CD = 7.88) threshold, indicating statistically significant differences. These findings indicate that the HLCA algorithm offers clear advantages over other protein complex algorithms in accuracy and comprehensive performance.

On the MIPS ground truth, the average rankings of the HLCA algorithm for F-measure, Accuracy, and F1 + ACC are 3.4, 1.2, and 1.0. Notably, it ranks first in Accuracy and F1 + ACC while maintaining a top position for F-measure. These results indicate that the HLCA algorithm maintains robust and stable performance across different datasets. In addition, the ranking differences observed under all three evaluation metrics exceed the critical difference (CD = 7.88) threshold, confirming that the performance improvements are statistically significant. The HLCA algorithm achieves consistent top rankings and statistically significant results across both benchmark datasets. These outcomes highlight its robustness and competitiveness in protein complex identification.

### 3.6 Enrichment analysis

To evaluate the effectiveness of the HLCA algorithm, Gene Ontology (GO) enrichment analysis is performed on the protein sets of all predicted complexes. The analysis covers three GO categories: biological process (BP), molecular function (MF), and cellular component (CC). For the Gavin1, Gavin2, K_extend, DIP, and BIOGRID datasets, at least 96.94%, 93.19%, 91.87%, 91.55%, and 91.41% of the predicted complexes, respectively, exhibit significant enrichment in at least one GO category. These results indicate that nearly all predicted complexes show strong biological relevance across at least one GO dimension. Six representative predicted complexes are further selected as case studies to demonstrate biological plausibility and functional coherence. Their detailed GO enrichment results are summarized in Table 2, where each complex shows significant enrichment across multiple GO terms. Overall, these findings confirm the biological validity of the protein complexes identified by HLCA.

**TABLE 2:** GO enrichment analysis of six predicted protein complexes.

ID	Biological process (BP)		Molecular function (MF)		Cellular component (CC)	
	GO term	P-value	GO term	P-value	GO term	P-value
1	intracellular sphingolipid homeostasis *GO:0009156*	$1.15\times 10^{-7}$	syntaxin binding *GO:0019905*	$1.14\times 10^{-2}$	GARP complex *GO:0000938*	$1.00\times 10^{-8}$
2	tRNA gene clustering *GO:0070058*	$6.01\times 10^{-12}$	chromatin binding *GO:0003682*	$1.35\times 10^{-3}$	condensin complex *GO:0000796*	$1.77\times 10^{-12}$
3	Golgi to plasma membrane transport *GO:0006893*	$2.7\times 10^{-10}$	—	—	exomer complex *GO:0034044*	$1.54\times 10^{-11}$
4	vesicle fusion with Golgi apparatus *GO:0048280*	$3.89\times 10^{-19}$	SNAP receptor activity *GO:0005484*	$1.82\times 10^{-15}$	SNARE complex *GO:0031201*	$1.47\times 10^{-17}$
5	maturation of SSU rRNA *GO:0030490*	$3.39\times 10^{-32}$	snoRNA binding *GO:0030515*	$5.49\times 10^{-18}$	90S preribosome *GO:0030686*	$2.34\times 10^{-32}$
6	RNA polymerase II preinitiation complex assembly *GO:0051123*	$8.63\times 10^{-34}$	transcription coregulator activity *GO:0003712*	$2.23\times 10^{-30}$	mediator complex *GO:0016592*	$9.83\times 10^{-45}$

**FIGURE 13:**
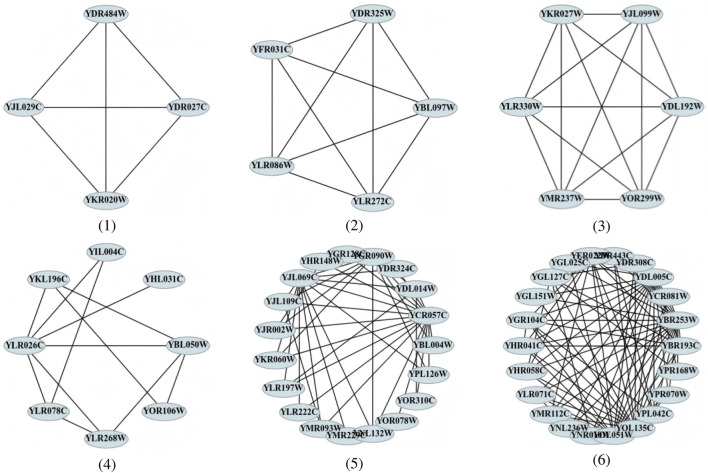
Examples of six predicted protein complexes.

Experimental results indicate that three predicted complexes shown in Figure 13 (1–3) correspond to known protein complexes. Specifically, complex (1) is enriched in the sphingolipid metabolic process and intracellular sphingolipid homeostasis with a highly significant P -value of $1.15\times 10^{-7}$ . Complex (2) is associated with tRNA gene transcription with $P=6.01\times 10^{-12}$ . Complex (3) shows significant enrichment in Golgi-to-plasma membrane transport with $P=2.47\times 10^{-10}$ , indicating involvement in well-characterized trafficking pathways.

The remaining three complexes in Figure 13 (4–6) represent novel predictions not present in standard reference datasets, yet they also exhibit strong GO enrichment and biological plausibility. For example, complex (4) is significantly enriched in Golgi vesicle fusion $\left(P=3.89\times 10^{-19}\right)$ and SNAP receptor activity $\left(P=1.82\times 10^{-15}\right)$ , suggesting a functional role in the SNARE complex. Complex (5) is enriched in ribosomal subunit rRNA maturation $\left(P=3.39\times 10^{-32}\right)$ and snoRNA-binding functions $\left(P=5.49\times 10^{-18}\right)$ , consistent with a pre-90S ribosomal complex. Complex (6) is enriched in RNA polymerase II preinitiation complex assembly $\left(P=8.63\times 10^{-34}\right)$ and transcriptional co-regulatory activity $\left(P=2.23\times 10^{-30}\right)$ , corresponding to the Mediator complex. Taken together, these examples demonstrate that HLCA is able to recover known protein complexes and, at the same time, predict novel complexes with strong functional relevance. The consistent and highly significant GO enrichment across BP, MF, and CC categories underscores the algorithm’s potential for biologically meaningful protein complex discovery.

## 4 Discussion and conclusion

Protein complexes are crucial for cellular processes and are key to understanding cellular functions. We propose a hypergraph learning and core-attachment strategy (HLCA) algorithm for protein complex identification. The method incorporates higher-order topological information and node metrics within the core-attachment framework. First, a hierarchical compression strategy recursively reduces the input hypergraph into smaller hypergraphs. Hypergraph convolution is applied to each smaller hypergraph to generate node representations at different granularities. These representations are integrated to capture higher-order topology. A weighted PPI network is then constructed based on similarity. Core clusters are extracted from the weighted PPI network. Nodes with the highest node metrics not yet in the core clusters are subsequently added. Experimental results show that using CYC2008 and MIPS as the ground truth, the HLCA algorithm achieves an average improvement of 25.77% and 21.47% in F1 + ACC. HLCA algorithm enhances the accuracy of protein complex identification by combining higher-order topology with network characteristics. This has spurred advancements in the fields of data mining and bioinformatics.

However, the current approach constructs hypergraphs using only protein-protein interactions without incorporating biological information. Integrating such biological information may further enhance identification performance.

## Data Availability

The datasets used in this study are publicly available from https://github.com/yyohu/HLCA.
